# Artificial Light at Night Affects Emergence from a Refuge and Space Use in Guppies

**DOI:** 10.1038/s41598-018-32466-3

**Published:** 2018-09-20

**Authors:** R. H. J. M. Kurvers, J. Drägestein, F. Hölker, A. Jechow, J. Krause, D. Bierbach

**Affiliations:** 1 Center for Adaptive Rationality, Max Planck Institute for Human Development, Lentzeallee 94, 14195 Berlin, Germany; 2 Leibniz-Institute of Freshwater Ecology and Inland Fisheries, Müggelseedamm 310, 12587 Berlin, Germany; 3 Institute of Biology, Freie Universität Berlin, Schwendenerstraße 1, 14195 Berlin, Germany; 4 Faculty of Life Sciences, Humboldt University, Invalidenstrasse 42, 10115 Berlin, Germany

## Abstract

Artificial light at night (ALAN) is a major form of anthropogenic pollution. ALAN is well known to affect different behaviours during nighttime, when changes in light conditions often have immediate consequences for the trade-offs individuals experience. How ALAN affects daytime behaviours, however, has received far less attention. Here we studied how ALAN affected daytime personality traits and learning ability. We exposed Trinidadian guppies, *Poecilia reticulata*, for 10 weeks to different ALAN levels: bright light (24 hrs bright light, ~5,000 lx), dim light (12 hrs bright light; 12 hrs dim light, ~0.5 lx) and control (12 hrs bright light; 12 hrs dark). Afterwards, we tested how the treatments affected diurnal emergence from a refuge, space use, activity, sociability and the ability to memorize the location of companion fish. Individuals exposed to the light treatments (both dim and bright light) emerged quicker from a refuge and fish from the bright light treatment spent relatively more time in the open area of the arena. ALAN did not affect any of the other behaviours, although memory could not be tested since fish did not learn the companions’ location. Our results demonstrate that ALAN, next to affecting nocturnal behaviours, can also affect key diurnal behavioural processes, associated with risk-taking.

## Introduction

The use of artificial light at night (ALAN) is a major form of anthropogenic pollution affecting a wide range of environmental processes^1–9^. The two main sources of light pollution are direct artificial light and skyglow. Direct light is usually high in intensity as it originates directly from street lighting, domestic and commercial light sources, or is reflected via surrounding surfaces^1^. Skyglow, on the other hand, is the portion of artificial light that is scattered and reflected back to Earth within the atmosphere^10,11^. Skyglow is a growing global phenomenon that is highly dynamic^12–14^. Compared to direct light, it is dim and spatially homogenous on a small scale, but can be bright compared to natural celestial light sources, such as stars or even moonlight^15^ and can alter nightscapes located far from urban areas^16,17^.

In terms of behavioural processes, ALAN can have distinct effects on the behaviour of animals during the nighttime^1^. Many species use light for orientation during nighttime navigation. ALAN can thus alter the movement trajectories of individuals, either because individuals are directly attracted to light (‘flight-to-light’) or because natural light sources used for navigation are masked (e.g., by skyglow). These effects have been observed in insects^18,19^, birds^20^, amphibians^21,22^, reptiles^23^ and fish^24^. Next to direct attraction, ALAN can alter the trade-offs animals face during the night. Especially, foraging benefits and predation risk can be strongly influenced by light conditions^1,25^. Approximately 30% of all vertebrates and more than 60% of all invertebrates are nocturnal, and for these organisms, their temporally differentiated niche has been promoted by highly developed senses^2^. Consequently, ALAN has different effects on diurnal and nocturnal species. Diurnal (and crepuscular) animals often become more night active with increasing ALAN (‘exploiting the night light niche’)^26–28^, whereas nocturnal animals often become less night active^29–32^.

Next to affecting behavioural processes during nighttime, ALAN can also alter behavioural processes during daytime. This has received especially attention in the context of activity patterns: ALAN has been shown to advance the onset of daily activity in European blackbirds, *Turdus merula*^33,34^, great tits, *Parus major*^26^ and to increase overall daily activity in zebra finches, *Taeniopygia guttata*^35^. Moreover, several studies, predominantly in rodents, have shown that ALAN can impair cognitive abilities such as spatial learning and memory. Rats and mice exposed to ALAN performed poorly in the Morris water maze^36–38^. In the diurnal rodent, the Nile grass rat, *Arvicanthis niloticus*, individuals exposed to three weeks of dim light during night showed impaired learning and memory in the Barnes maze^39^. In zebra finches, constant ALAN also resulted in impaired learning and cognition^35^. In contrast, in peafowls, *Pavo cristatus*, one-night light exposure did not affect problem-solving success^40^.

Although the effects of ALAN on daytime behaviour have received some attention, most notably in the context of (the onset of) activity and cognition, the extent to which ALAN affects other important behavioural traits such as risk-taking or sociability, has received very little attention (but see^35^). Such personality traits that are known to differ consistently among individuals can be linked to important life history traits of individuals, including survival and reproductive success^41–43^. Thus, to understand how ALAN affects a broader range of personality traits can be important to further increase our understanding of the potential impact of ALAN on animal populations. Here we studied how ALAN affects several such personality traits, including emergence, activity, space use, sociability and memory ability in the Trinidadian guppy, *Poecilia reticulata*.

Animals living in aquatic habitats, including fish, are by no means exempt from light pollution^6,24,44^. Skyglow, streetlights, floodlights at harbours and (fishing) boats can emit light onto the water surface that reaches higher intensities than the light of the natural full moon light^45^ and can be detected even off-shore in marine ecosystems above^46,47^ and under water^48^. ALAN might not only alter fish communities^49^ and physiology^50^, but also fish behaviour^51^. Several studies have shown that fish become disoriented when swimming near lights^24,52^. In Atlantic salmon, *Salmon salar*, ALAN disrupted the timing of migration (and thereby the social synchrony)^53,54^. ALAN may also affect schooling behaviour because vision is often crucial for communication between school members^55,56^. In walleye pollocks, *Theragra chalcogramma*, neighbour distance increased with decreasing light levels until it became too dark and schooling stopped^57^.

To investigate the effects of ALAN on a range of important personality traits, we exposed guppies to different ALAN treatments: bright light (24 hrs bright light, ~5,000 lx), dim light (12 hrs bright light; 12 hrs dim light, ~0.5 lx) and control (12hrs bright light; 12 hrs dark). Light treatments took place for 10 weeks after which we repeatedly tested fish during the day on emergence, activity, space use, sociability and their ability to memorize the location of companion fish in a T-maze. We expected that ALAN would lead to reduced memory-based performance in the T-maze. In terms of behavioural traits, we had no a priori expectations since research linking ALAN to personality traits is largely absent.

## Methods

### Study organisms and maintenance

We used captive adult Trinidadian guppies, which originated from wild guppies collected from the upper Arima River in Trinidad. Guppies have been bred in the lab for about 25 generations. We haphazardly caught individuals from a large mixed-sex stock tank containing hundreds of guppies. We then sexed and size-measured fish (standard length [SL] to the nearest mm) by taking a photo of the fish on scale mm paper that was subsequently analysed in ImageJ 1.49 v. Afterwards, fish were given a unique tag using Visible Implant Elastomer tags^58,59^. This enabled us to recognize individuals throughout the experiment while keeping them together in a group. We then randomly assigned fish to a holding tank with the constraint of a maximum of five males and five females per holding tank. In each light treatment (see below), we used three identical holding tanks with each tank containing five adult males and five adult females after tagging, resulting in 30 fish per treatment and 90 fish in total. Following tagging, the light treatments started. Fish were kept under the different light regimes for 10 weeks (August – October 2015), after which the behavioural testing started. Final sample size per treatment was lower than 30 since some fish lost their tag and were thus unidentifiable while a few others died. Final sample size: control: n = 20; dim light: n = 19, bright light: n = 23. During the light treatments and the behavioural testing, fish were fed twice daily (at 9:00 and 15:00) with TetraMin dry food.

### Light treatments

**Figure 1:**
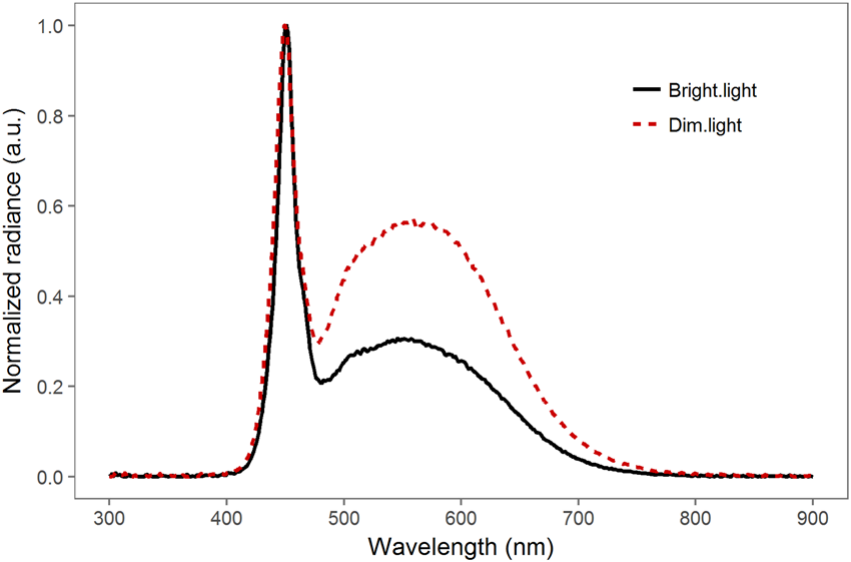
The light spectra of the bright and dim light conditions, showing spectral radiances normalized to the emission peak. The LEDs had a strong emission peak in the blue spectrum near 450 nm (typical for GaN based LEDs) and a broadband emission centred at a wavelength of 560 nm (yellow-green spectrum; originating from the phosphor layer). In the dim light condition, the spectrum altered in the way that the ratio of the blue peak and the broadband peak changed. At bright light conditions (black solid line), the fraction of blue emission (and therefore the colour temperature) was higher than when dimmed (red dashed line). This mimics the change from a mid-day light spectrum to an evening spectrum. The light spectrum for the dimmed treatment was closer to real-world street lighting or skyglow from LEDs.

We used three light treatments: (i) control: natural day-night rhythm of 12 hrs bright light (~5,000 lx) and 12 hrs dark (0 lx), (ii) dim light: 12 hrs bright light (~5,000 lx) and 12 hrs dim light (~0.5 lx) and (iii) bright light: 24 hrs of bright light (~5,000 lx). We decided on these light levels, since we wanted to study the effects of a low, but realistic, ALAN level (dim), and the effects of a very high ALAN level (bright), as compared to a control situation (control). The different treatments were set up in separate compartments next to each other in the same room. In each treatment, we used three holding tanks (60 × 30 × 30 cm) placed next to each other. Ambient light from neighbouring treatments and the surroundings was shielded. As lighting, we used dimmable light-emitting diodes (LEDs) emitting white light. Each compartment had four rows of LED lighting, placed above the holding tanks. The lights were controlled via time switches. In the control treatment, the LEDs were fully switched on for 12 hrs (day) and switched off for 12 hrs (night). In the dim light treatment, the LEDs were fully switched on for 12 hrs (day) and dimmed to ca. 0.5 lx for 12 hrs (night). Lights switched automatically at 6 am (‘night to day’) and 6 pm (‘day to night’). In the bright light treatment, the LEDs were fully switched on for 24 hrs (day + night). To confirm that our treatments indeed resulted in different light conditions, we used an ILT1700 Research Radiometer (range: 0.00167–1,670,000 lx, International Light Technologies, Peabody, MA) to measure light intensity. During the daylight period, we took 15 measurement points (arranged in a grid at the bottom of the tank) for each of the nine tanks by placing the SUD033/Y/W Underwater Broadband Silicon Detector (400–700 nm, photopic calibration) at the bottom of the tanks. The average daylight intensity for the three treatments were: control: mean ± SE = 4,607 ± 165 lx; dim light: 4,652 ± 143 lx; and bright light: 5,202 ± 165 lx. The average night-light intensity for the three treatments were: control: mean = 0 lx (Limited by the measurement range of the instrument. The light level was measured during the day to be below 0.035 lx, indicating a good level of shielding from ambient light.); dim light: 0.55 lx; and bright light: 5,202 lx. The spectra of the LEDs was measured using a spectro-radiometer with a measurement range of 250–1000 nm and 4.5 nm wavelength resolution (JETI Specbos 1211 UV, Jena Technische Instrumente, Jena, Germany). The LEDs had a strong emission peak in the blue spectrum near 450 nm (typical for GaN based LEDs) and a broadband emission centred at a wavelength of 560 nm (yellow-green spectrum; originating from the phosphor layer; Fig. 1). In the dim light condition, the spectrum altered in the way that the ratio of the blue peak and the broadband peak changed.

### Behavioural tests

**Figure 2:**
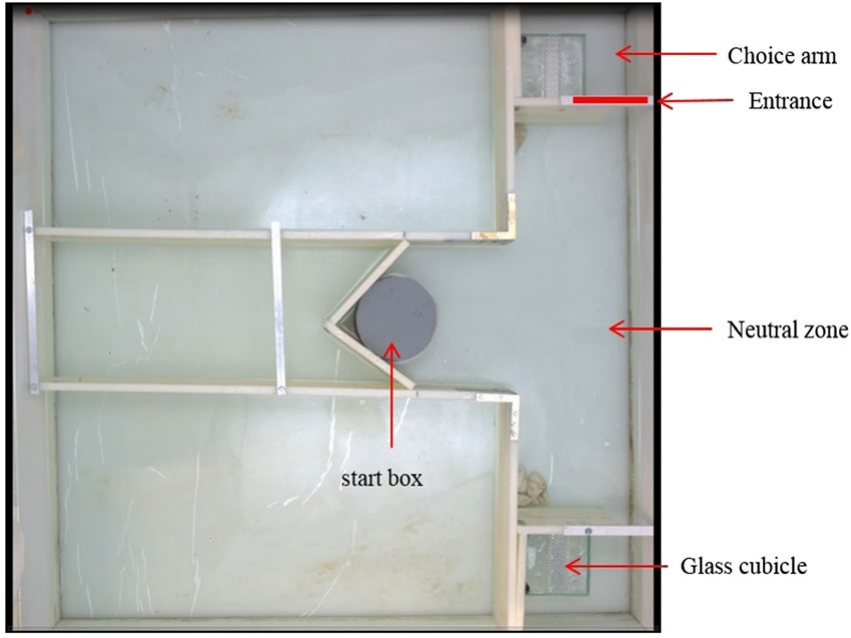
Image of the experimental arena (88 × 88 cm) used for testing the effects of ALAN on guppy personality traits and memory ability. A guppy entered the T-shaped arena via the start box. The start box contained a piece of foam, which was removed at the start of a trial allowing fish access to the neutral zone. We placed landmarks (stones) in the corners of the neutral zone to help fish orient and distinguish between the left and right side. These landmarks were always positioned in the same way. From the neutral zone, fish could enter two choice arms, each of which contained a glass cubicle. One of the glass cubicles contained a shoal of four companion fish whereas the other one was empty.

To test the effect of the light treatments on the personality traits and memory ability, we used a T-maze (Fig. 2). The T-maze consisted of (i) a start box: the box from which the fish was introduced, (ii) a neutral zone: the zone the fish entered after leaving the start box, but before choosing one of the two choice arms, and (iii) two choice arms, and was filled with 10 cm of aged tap water of 26 °C. A fish was considered to enter a choice arm when its entire body crossed the entrance line of that choice arm. Each choice arm contained a glass cubicle (8 × 8 × 8 cm), which was either empty or contained four companion fish. As companion fish, we used two unfamiliar females and males and we exchanged companion fish between each subsequent trial of a focal fish (i.e., fish never experienced the same companions twice). The focal fish could not see the companion fish from the neutral zone but had to enter the choice arm to inspect the possible presence of the companions. We thus used the presence of companions as reward. This procedure was successfully used before in testing the learning ability of different fish species, including guppies^60–62^. We did not use food as a reward since guppies frequently ignore food rewards in choice arenas because they can live multiple days without food.

The behavioural tests lasted three weeks (26.10.2015–15.11.2015). The different light treatments continued during this period, but we performed all behavioural tests during daytime (between 9 am and 5 pm). Each week, we tested the fish from one tank of each of the three treatments (thus a maximum of 30 tested fish per week). We tested each fish twice a day for a period of five days, resulting in 10 trials per fish and 620 trials in total. For every trial, the fish were haphazardly caught from their holding tank and transferred to the start box. After 1 min of acclimatization, we gently removed the foam blocking the entrance of the start box, allowing the fish access to the neutral zone. If the fish did not emerge from the start box after five minutes, we removed the lid covering the start box. If the fish did not emerge after an additional three minutes, we gently removed the entire starting box, though this happened rarely (20 out of 620 trials). Trials ended 1 min after the focal fish discovered the companions or 10 min after emergence, if the focal fish did not enter the choice arm with the companions (which happened in 40 out of 620 trials). The side of the companion fish (i.e., left or right) was the same for all fish from one holding tank, but alternated between fish from different holding tanks, to control for a possible side bias. An overhead camera recorded all trials. From the recordings, we scored the following behaviours: *emergence time*: the time before the fish emerged from the starting box (max = 480 s). *Edge time:* the proportion of time spent within 2 cm of the wall of the tank or the starting box. *Activity:* the mean velocity in cm per s. *Edge time* and *activity* were calculated during the first min after emergence or shorter if the fish entered one of the two choice arms within one minute. We used the program Ethovision XT 10.1 (Noldus) for tracking and calculating the times and velocity of fish. *Decision time:* the time taken after emergence, to cross one of the two entrance lines to the choice arms. If a fish did not enter either one of the choice arms within 10 min, it received a decision time of 600 s. *Social time*: the time spent with the companions after they were encountered (max: 60 s). If a focal fish did not enter the choice arm containing the companions, its social time was treated as a missing value. Furthermore, we scored for each trial whether the focal fish made the correct (i.e., side of the companions) or incorrect decision. Again, if a fish did not enter either of the choice arms within 10 min this was treated as a missing value.

### Statistical analysis

For all modelling procedures (except edge time, see below) we used the Bayesian modelling package MCMCglmm for R^63^, using flat uninformative Gamma priors. We used conservative long iteration chains, consisting of 1,030,000 iterations, a thinning interval of 1,000 and a burn-in phase of 30,000. We visually confirmed convergence using the plot function for MCMCglmm. We fitted separate mixed-effect models for the following response variables: emergence time (log-transformed), activity, decision time (log-transformed), social time and decision correct (yes/no). For emergence time, activity and decision time, we fitted a Gaussian distribution. For decision correct (yes/no) we used a “categorical distribution”. Social time was heavily skewed towards values of 60 s. Therefore, we transformed these data into a binary variable with “1” implying that the focal fish stayed the entire 60 s with the companions after discovering them and “0” implying that the focal fish left the companions after discovering them (i.e., all values less than 60 s). As fixed effects, we fitted in all models light treatment, trial number, the interaction between light treatment and trial number, sex, body size and the side of the companion fish (left/right). Individual was used as a random term (tank did not explain any variation in the models and was therefore not included in the random effect structure). We performed backward selection using nonzero overlapping CIs and *P* values (<0.05). The significance of the fixed effects was calculated using the pMCMC and we report post.mean and 95% lower and upper CI values. We additionally calculated repeatability estimates (*r*) and associated 95% CIs for each response variable based on the posterior distribution from the most parsimonious Bayesian mixed models. In all of these most parsimonious models, significant fixed effects were present and we report the adjusted repeatability (adj. *r*) values of these models including the significant fixed effects^64^. Significance of repeatability was based on nonzero overlapping CIs.

Since edge time is expressed as proportional data, we used the glmer function (rather than the MCMCglmm) from the lme4 package^65^, using a binomial link function. The random and fixed effect structure were the same as described above, and we again performed a backward model selection procedure. We performed all statistical procedures in R (Version 3.4.4).

### Ethical permission

Experiments reported in this study were carried out in accordance with the recommendations of “Guidelines for the treatment of animals in behavioural research and teaching” (published in Animal Behavior 1997) and comply with current German law approved by LaGeSo Berlin (G0117/16 to D.B.).

## Results

**Figure 3:**
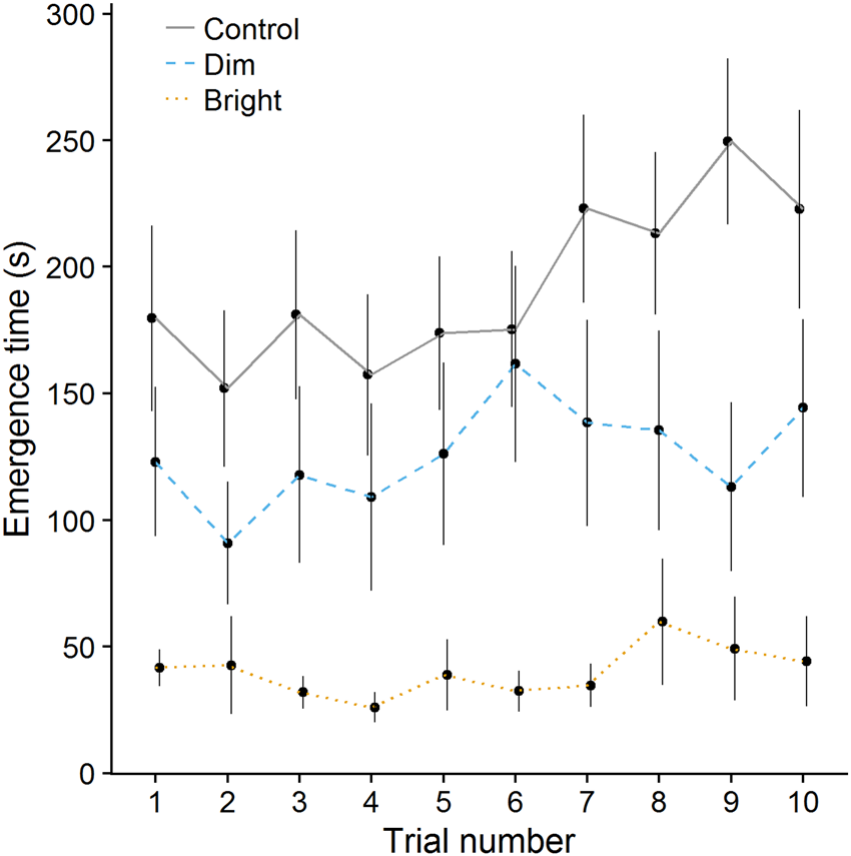
The effect of trial number on the average emergence time for fish from the three light treatments (i.e., control, dim light and bright light). Overall, fish from the control treatment had longer emergence times as compared to the fish from the bright and dim light treatments. Moreover, the emergence time increased over the course of the experiment for control fish, but not for fish from the dim or bright light treatments. Errors bars represent standard error.

**Figure 4:**
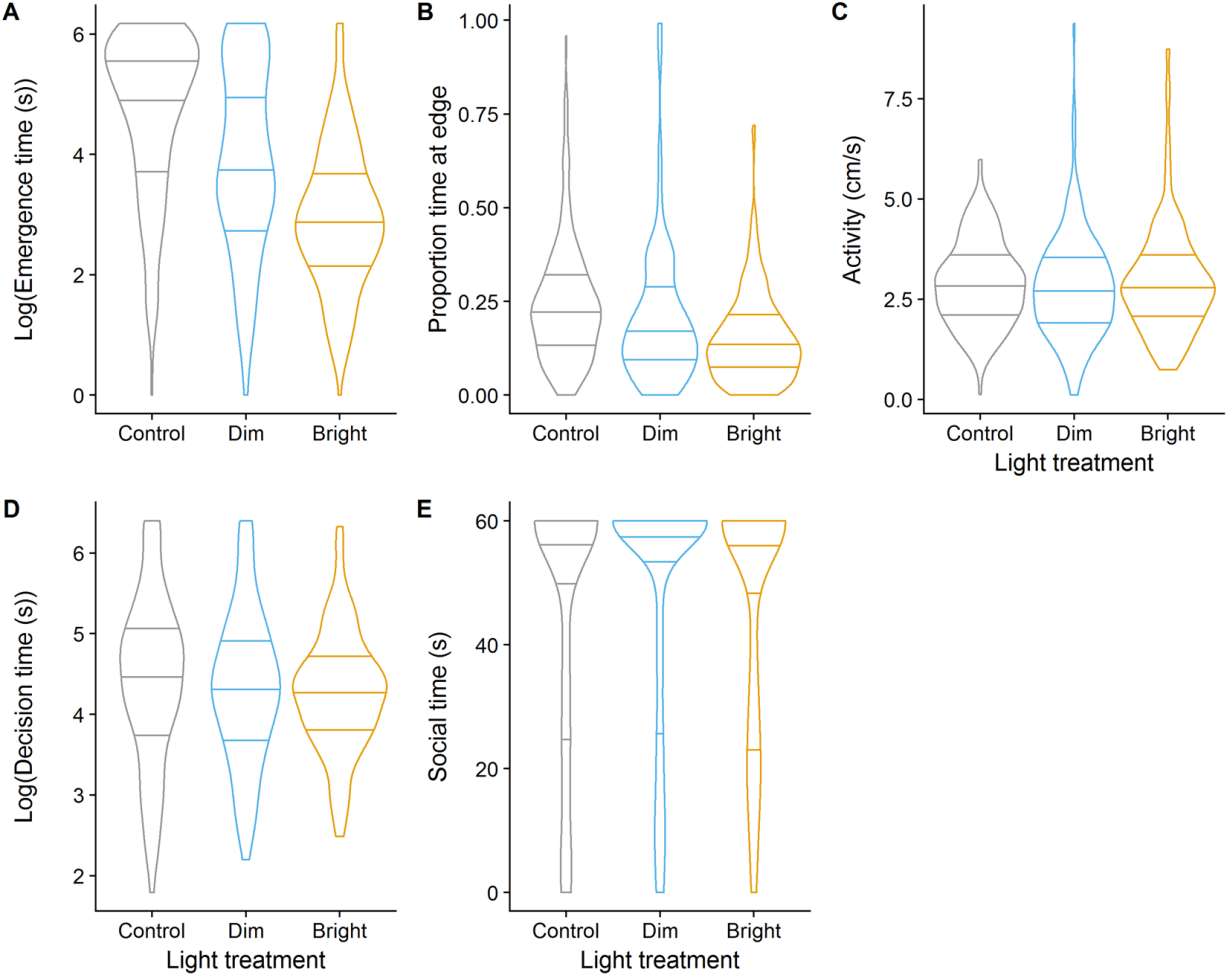
The effect of the three light treatments (i.e., control, dim and bright light) on (**A**) emergence time, (**B**) edge time (**C**) activity, (**D**) decision speed, and (**E**) social time. (**A**) Fish had shorter emergence times in the dim and bright light treatments as compared to fish from the control treatment. (**B**) Fish from the bright light treatment spent less time close to the edge of the arena than control fish. (**C**–**E**) There were no significant differences between treatment groups in activity, decisions speed or social time. Horizontal lines in violin plots display mean and interquartile range.

There was a significant interaction between light treatment and trial number on emergence time (interaction control*bright light treatment: post.mean [CI_lower_, CI_upper_] = −0.08 [−0.13, −0.03], *P* < 0.001; interaction control*dim light treatment: −0.03 [−0.09, 0.02], *P* = 0.266). Fish from the control treatment emerged slower over the course of the experiment (effect of trial number in control fish: 0.06 [0.03, 0.10], *P* < 0.001; Fig. 3). However, for fish exposed to dim and bright light condition at night there was no significant effect of trial number on emergence time (both *P* > 0.2; Fig. 3), suggesting that fish experiencing ALAN did not adjust their emergence time over the course of the experiment. Removing the interaction term from the model showed that fish from the dim and bright light condition had, overall, shorter emergence times as compared to fish that were exposed to dark nights (bright light: −1.73 [−2.46, −1.16], *P* < 0.001; dim light: −0.71 [−1.39, −0.05], *P* = 0.022; Fig. 4A). There was an effect of body size with smaller fish emerging quicker (0.09 [0.03, 0.15], *P* = 0.004; Suppl. Fig. 2A) and no effect of sex or side of the companions (both *P* > 0.2). Emergence time showed high repeatability (adj *r* [CI_lower_, CI_upper_] = 0. 61 [0.52, 0.71]).

**Figure 5:**
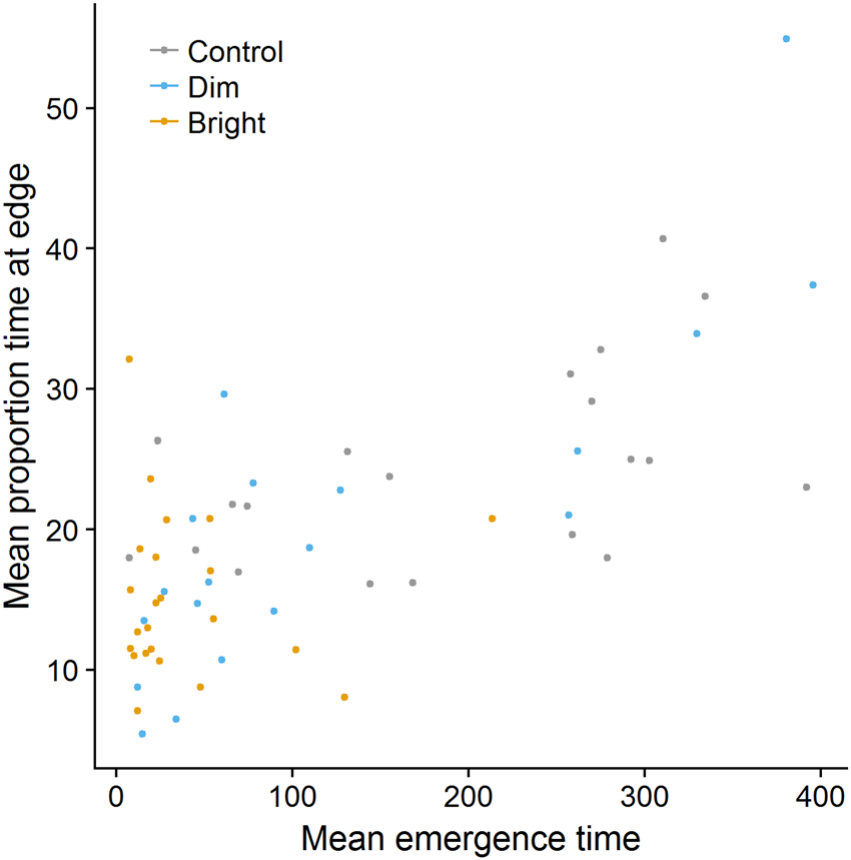
There was a positive relationship between an individual’s average emergence time and average proportion of time spent close to the edge of the arena.

We did not observe a significant interaction between light treatment and trial number on edge time (both *P* > 0.2). Fish from the bright light treatment spent less time close to the edge than fish from the control treatment (estimate ± SE = −2.16 ± 0.76, z = −2.83, *P* = 0.005; Fig. 4B). There was no difference between fish from the dim light and control treatments (*P* > 0.2). There was no effect of body size, side of the companions (both *P* > 0.2) or sex (*P* = 0.083) on edge time. There was a positive effect of trial number on edge time (estimate ± SE = 0.17 ± 0.07, z = 2.28, *P* = 0.023). When correlating an individual’s average emergence time with edge time, we found a positive correlation (Spearman’s rho = 0.61, *P* < 0.001; Fig. 5), implying that fish emerging, on average, relatively late also spent a relatively large amount of time close to the wall.

We did not observe a significant interaction between light treatment and trial number on activity (both *P* > 0.2) nor a significant main effect of light treatment (both *P* > 0.2; Fig. 4C). There was a significant effect of sex on activity (−0.64 [−1.09, −0.22], *P* = 0.004; Suppl. Fig. 1C) with females showing higher average speed than males and a negative effect of trial number on activity (−0.04 [−0.07, −0.02], *P* < 0.001). There was no significant effect of body size (*P* > 0.2) or the side of the companions (*P* = 0.094) on activity. Activity was significantly repeatable (adj *r* [CI_lower_, CI_upper_] = 0. 45 [0.35, 0.55]).

Likewise, there was no significant interaction between light treatment and trial number on decision speed (both *P* > 0.2) nor a significant main effect of light treatment (both *P* > 0.15; Fig. 4D). Females made faster decisions than males (0.42 [0.14, 0.68], *P* = 0.006; Suppl. Fig. 1D). There was no effect of body size, trial number or side of the companions on decision speed (all *P* > 0.1). Decision speed was significantly repeatable (adj *r* [CI_lower_, CI_upper_] = 0. 27 [0.19, 0.37]).

Also for social time, there was no significant interaction between light treatment and trial number (both *P* > 0.2) nor a significant main effect of light treatment (both *P* > 0.1, Fig. 4E). There was an overall negative effect of trial number on social time (−10.15 [−17.55, −3.35], *P* < 0.001) implying that fish shoaled less with companion fish over the course of the experiment. Males were more likely to shoal with the companion fish after discovery than females (54.38 [1.81, 111.17], *P* = 0.028; Suppl. Fig. 1E). There was no effect of body size or side of the companions on social time (all *P* > 0.2). Social time was significantly, albeit lowly, repeatable (adj *r* [CI_lower_, CI_upper_] = 0. 21 [0.11, 0.34]).

**Figure 6:**
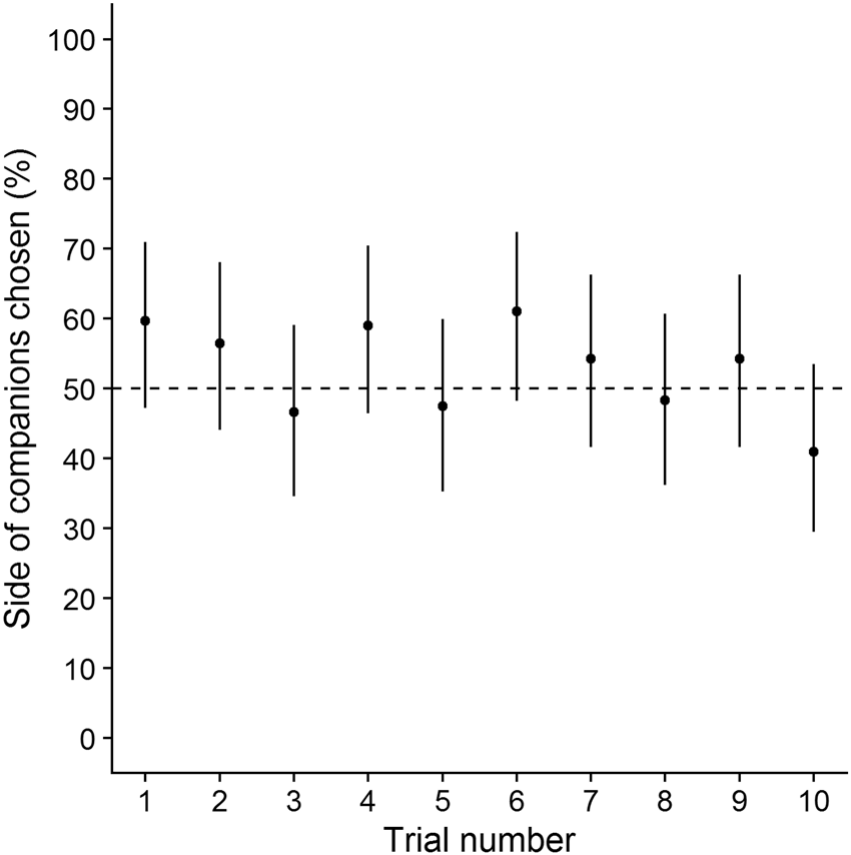
The effect of trial number on the likelihood of fish choosing the side containing the companion fish (“decision correct”). There was no effect of trial number (i.e., experience) on the likelihood of fish to choose the side containing the four companion fish. The dashed line shows the chance expectation. Error bars indicate 95% CIs.

We did not find a significant interaction between light treatment and trial number on correct decisions (both *P* > 0.2) nor a significant main effect of light treatment (both *P* > 0.1). Moreover, there was no significant effect of trial number on the likelihood of fish choosing the side containing the companion fish (*P* = 0.058; Fig. 6) suggesting that fish either did not memorize the location of the companion fish from previous experiences or were not motivated to visit the companion fish. There was no effect of sex or body size on the likelihood of choosing the side with the companions (both *P* > 0.2). There was a significant effect of the side of the companions on the likelihood of choosing the side containing the companion fish (76.38 [13.01, 142.26], *P* < 0.001): when the companion fish were positioned on the right side, the focal fish were more likely to choose the side with the companions than when the companion fish were positioned on the left side. This was, however, entirely due to a side-bias of the focal fish.

## Discussion

The light at night treatments affected the diurnal behaviours of our focal fish in two ways. First, fish exposed to ALAN emerged quicker from the refuge than control fish. And whereas control fish increased their emergence time with repeated exposure, fish exposed to ALAN did not change their emergence time over the course of the experiment. Second, fish from the bright light treatment spent less time close to the wall of the arena and more time in the open (riskier) part of the arena than control fish. Importantly, the dim light treatment had a relatively low level of light intensity (~0.5 lx), only slightly above full moon light conditions and in the range of urban skyglow for overcast nights^15^. Fish from the bright light treatment showed a stronger reduction in emergence time than fish from the dim light treatment (Fig. 3). Most studies on ALAN compare only two ALAN levels (ALAN versus no ALAN), but the effects of ALAN on behavioural and ecological processes are in most cases not binary, but gradual, and recent studies call for more research on such dose dependent effects by considering a wider range of (realistic) ALAN levels^9^. Although our study only included three levels of ALAN and firm conclusions on such dose dependent effects may require a larger range of ALAN levels (e.g.^26^), our results nonetheless suggest that increased levels of brightness may be associated with reduced emergence time in a dose dependent manner.

Faster emergence and increased use of open areas, most likely, would increase the risk of an individual. When startled, guppies quickly flee into hiding, searching the cover of floating leaves or small rock crevices. In our experiment, the cover of the start box is likely to serve a similar purpose, and quickly leaving the start box can thus be seen as an increased propensity to take risks, in line with previous studies using emergence time to quantify risk taking in this species^66^ and other poeciliids^67,68^. It is possible that ALAN increased activity levels at night and associated metabolic costs thereby altering the trade-off between foraging and risk during daytime, forcing hungrier animals to prefer food over safety. Harris *et al*. showed that guppies from high predation sites emerged sooner from shelter than guppies from low predation sites^66^. Similar results were found in other poeciliids species^67,68^, and repeatedly chasing fish with a net resulted in increased risk taking. Interestingly, more active, bold, and exploratory guppies survived longer when exposed to a piscivorous predator in a laboratory experiment^69^. Higher risk taking in guppies thus seems, at least partly, to be an adaptive response to elevated dangerous and stressful environments. Our light treatments may have induced similar increased stress levels, resulting in increased risk taking. However, an alternative explanation is that fish in the (dark) control treatment, may have developed a preference for darker regions (as compared to fish from the light treatments), which would also result in longer emergence time and more time close to the wall. To disentangle both explanations, further risk taking tests independent of light conditions (e.g., startle test) would be required. Nonetheless, in both scenario’s, ALAN would lead to an increase in risk taking.

Activity showed significant repeatability, but was not affected by the light treatments. Previous studies have shown that ALAN can advance the onset of daily activity^26,33,34^ but the effects on average activity during the day are less clear. Earlier work has reported that ALAN either increased^35^ or decreased daytime activity^26^. However, comparing the effects of ALAN on activity patterns during day- and nighttime separately suggests that the effects on nighttime activity are substantially larger^26^ so we might expect guppies to show stronger activity responses to ALAN during nighttime. Likewise, we did not find an effect of ALAN on daytime sociability. We had no a-priori expectations regarding sociability as little to no studies have investigated this relationship. In fact, the consequences of ALAN on social and group processes is largely unknown^70^. Future studies quantifying the degree (and onset) of activity and sociability both during day and nighttime and over longer periods, would be required to obtain a more complete picture of how ALAN affects such important behavioural processes.

Over the course of the experiment, guppies did not develop a preference for the choice arm containing the companion fish, implying that fish were either unable to learn the location of the companion fish, or they simply lacked the motivation to shoal with conspecifics. The latter is suggested, at least partly, by an overall decrease in sociability over the course of the experiment. Burns and Rodd found that guppies were able to perform above chance level in an arena containing an empty arm and an arm with conspecifics^60^. However, they used only males as focal fish and females as conspecifics exploiting the well-known drive of male guppies to search for mating opportunities^71^. In our experiment, we also found that, once discovered, males were more likely to shoal with their companions than females (Suppl. Fig. 1E), suggesting that this experimental design is indeed better suited for male than female guppies. Given the overall absence of any learning, we were thus not able to test the effect of ALAN on learning ability.

Finally, there was a slight difference in average daytime light levels between the three treatments, with the light level of the bright light treatment (±5,202) being slightly higher than the dim (±4,652) and control (±4,607) treatment. Though we cannot exclude that this may have had some effect, the relative difference in light levels between the treatments during the day is very small (±10%) as compared to the relative difference in light levels during the night. It thus seems reasonable to assume that our observed behavioural differences are the result of nighttime differences in light regime.

To conclude, we have shown that ALAN reduced daytime emergence time and increased the time spent in the open in guppies. Moreover, whereas control fish increased their emergence time over repeated exposures; fish from the light treatments did not adjust their emergence time over repeated exposure. ALAN did not affect daytime activity, sociability or decision speed. Our results demonstrate that ALAN, next to affecting nocturnal behaviours, can also affect daytime behavioural processes, associated with risk-taking.

## Electronic supplementary material

Supplementary Material

Dataset 1

## Data Availability

All behavioural data are included as Supplementary Information.
