# Supplementary material for: Artificial Light at Night Affects Emergence from a Refuge and Space Use in Guppies
Source: Sci Rep. 2018 Sep 20;8:14131. doi: 10.1038/s41598-018-32466-3 (PMC6147999; doi:10.1038/s41598-018-32466-3)
Supplement: Supplementary file 1 — Supplementary Material [file 41598_2018_32466_MOESM1_ESM.docx]

Supplementary Material Belonging to

**Artificial Light at Night Affects Emergence from a Refuge and Space Use in Guppies**

Kurvers RHJM, Drägestein J, Hölker F, Jechow A, Krause J & Bierbach D


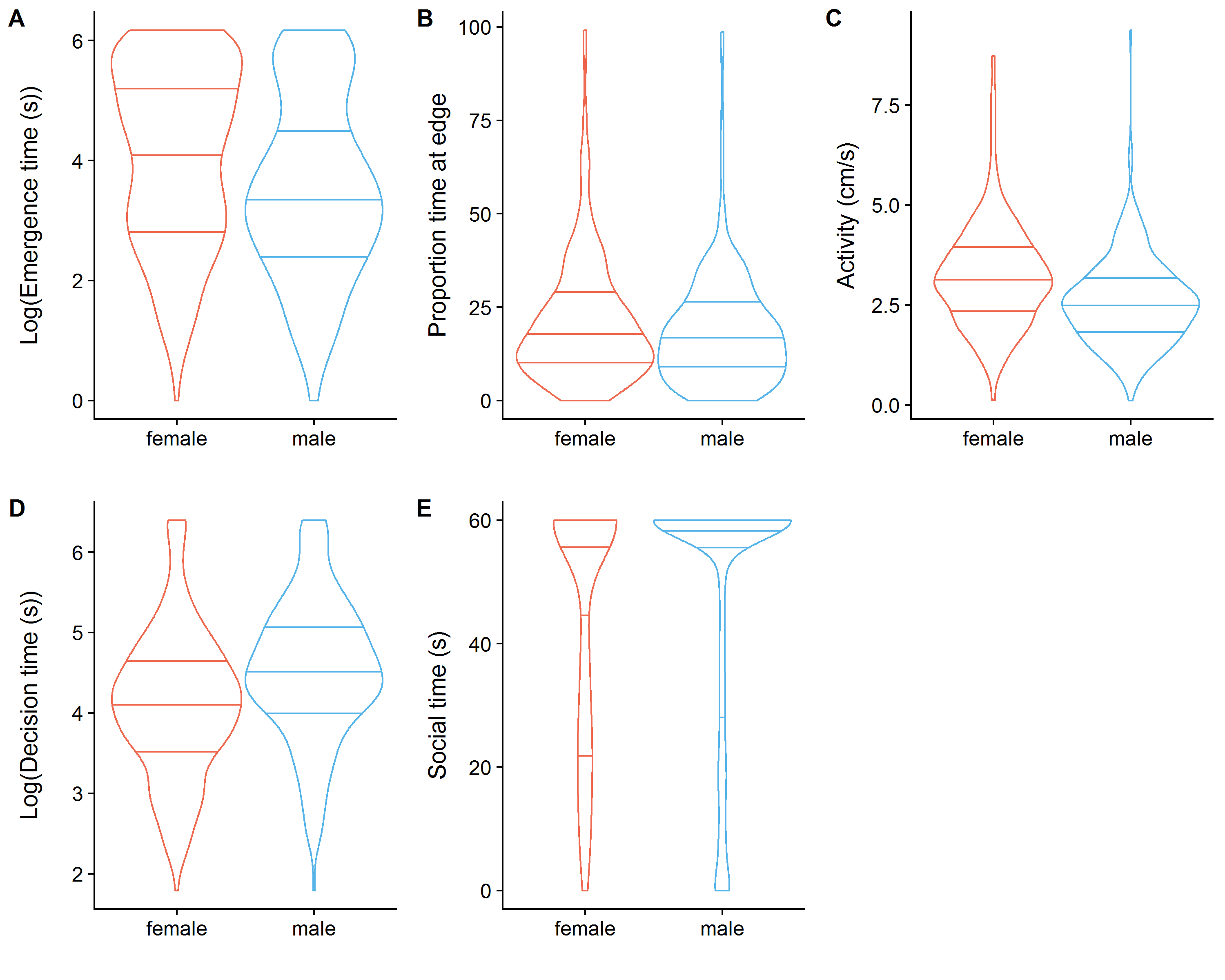


**Supplementary Figure 1.** The relationship between sex and (A) emergence time, (B) edge time, (C) activity, (D) decision time, and (E) social time. Females had (C) higher activity levels than males, (D) and a lower decision time, and (E) a lower social time. Horizontal lines in violin plots display mean and interquartile range. For statistical details, see main text.


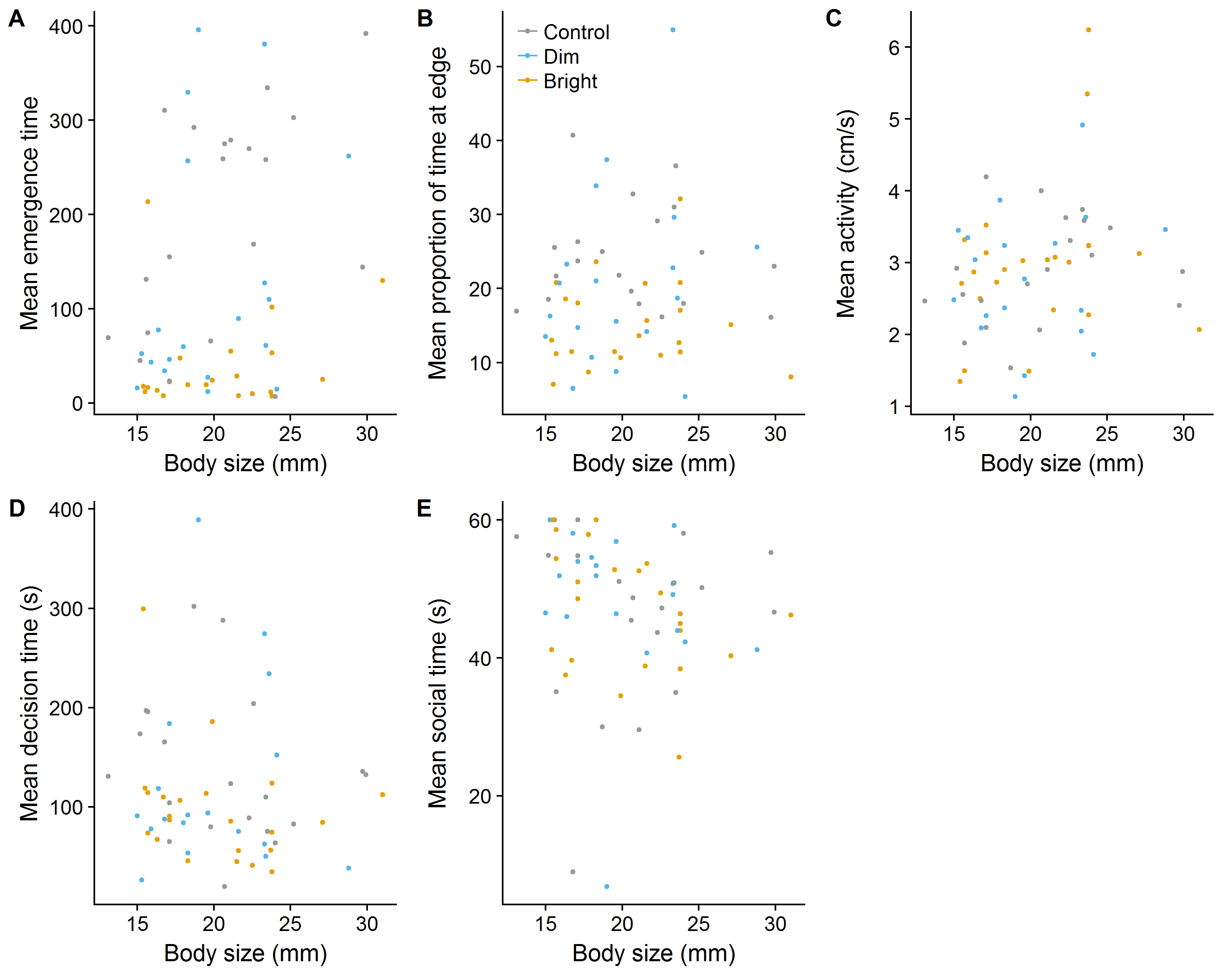


**Supplementary Figure 2.** The relationship between body size and mean (A) emergence time, (B) edge time, (C) activity, (D) decision time, and (E) social time. (A) Only the relationship between body size and emergence time was significant, with smaller fish emerging, on average, quicker than larger fish. For statistical details, see main text.
